# Circadian-period variation underlies the local adaptation of photoperiodism in the short-day plant *Lemna aequinoctialis*

**DOI:** 10.1016/j.isci.2022.104634

**Published:** 2022-06-17

**Authors:** Tomoaki Muranaka, Shogo Ito, Hiroshi Kudoh, Tokitaka Oyama

**Affiliations:** 1 Faculty of Agriculture, Kagoshima University, Korimoto 21-24-1, Kagoshima 890-0065, Japan; 2 Department of Botany, Graduate School of Science, Kyoto University, Kitashirakawa-Oiwake-cho, Sakyo-ku, Kyoto 606-8502, Japan; 3 Center for Ecological Research, Kyoto University, Hirano 2-509-3, Otsu 520-2113, Japan

**Keywords:** Plant biology, Plant Biology, Plant genetics

## Abstract

Phenotypic variation is the basis for trait adaptation via evolutionary selection. However, the driving forces behind quantitative trait variations remain unclear owing to their complexity at the molecular level. This study focused on the natural variation of the free-running period (FRP) of the circadian clock because FRP is a determining factor of the phase phenotype of clock-dependent physiology. *Lemna aequinoctialis* in Japan is a paddy field duckweed that exhibits a latitudinal cline of critical day length (CDL) for short-day flowering. We collected 72 strains of *L. aequinoctialis* and found a significant correlation between FRPs and locally adaptive CDLs, confirming that variation in the FRP-dependent phase phenotype underlies photoperiodic adaptation. Diel transcriptome analysis revealed that the induction timing of an *FT* gene is key to connecting the clock phase to photoperiodism at the molecular level. This study highlights the importance of FRP as a variation resource for evolutionary adaptation.

## Highlights

• Natural variation of flowering/circadian traits in a paddy-field duckweed is studied.
• Critical day length for flowering of the duckweed in Japan shows a latitudinal cline.
• A negative correlation between critical day length and circadian period was found.
• An *FT* gene responding to lengthening of the dark period was isolated.

## Graphical abstract

**Figure:**
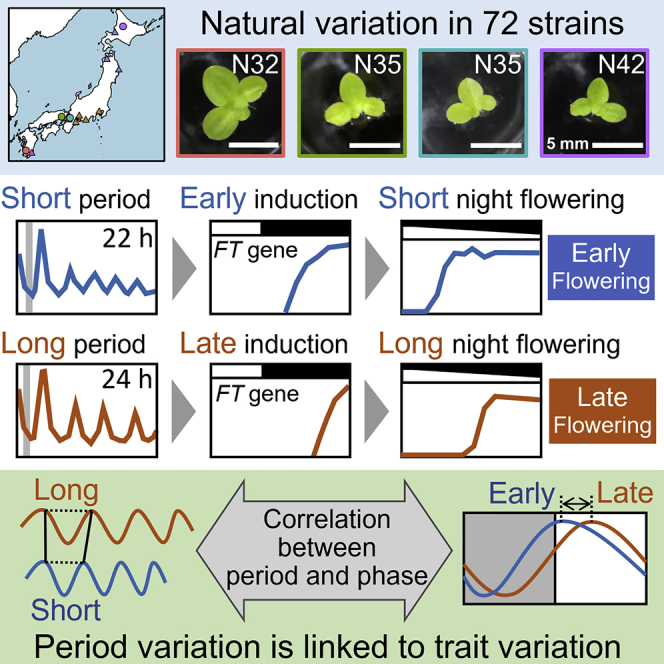

## Introduction

Understanding the molecular framework underlying the phenotypic changes in local adaptation is an important goal in evolutionary ecology. Natural variation in phenotypic traits plays a key role in evolutionary adaptation (Barret and Schluter, 2008; Alonso-Blanco et al., 2009; Matuszewski et al., 2015). However, the molecular mechanisms that generate such phenotypic variations in natural settings are still poorly understood because they are complex traits at the molecular level (Boyle et al., 2017; Sella and Barton, 2019; Feiner et al., 2021).

The circadian clock is an endogenous timing system that generates a self-sustained daily oscillation entrained to day-night cycles via environmental cues and regulates various physiologies to optimize the timing of development, stress responses, and metabolism (Pittendrigh 1960; Greenham and McClung, 2015). The eukaryotic circadian clock comprises feedback loops of multiple clock genes (Saini et al., 2019). The free-running period (FRP) of the circadian clock is a polygenic trait that exhibits natural variations (Michael et al., 2007; Brown et al., 2008; Pivarciova et al., 2016; Salmela and Weinig, 2019). The clock with an FRP deviating from 24 h is entrained to the 24-h day-night cycles (Johnson et al., 2003). The peak timing (phase) of an entrained rhythm also shows a natural variation. Previous studies suggest that diel phase determination is related to FRP in various organisms; a longer FRP results in a phase delay phenotype (Aschoff and Pohl, 1978; Rémi et al., 2010; Graf et al., 2010; Granada et al., 2013). Because the clock phase is relayed to physiological outputs, we hypothesized that FRP variation is associated with the local adaptation of the phase phenotype of clock-dependent physiology (Lankinen 1993; Dominoni et al., 2013; Helm et al., 2017).

FRP variation within 20–28 h has been reported in several plant species. In *Arabidopsis thaliana*, FRP showed a latitudinal cline and quantitative trait loci, including several *PSEUDO-RESPONSE REGULATOR* (*PRR)* genes, which encode a core component of the circadian clock, were detected (Michael et al., 2003). In Swedish *A. thaliana* accessions, an allele of the *COLD REGULATED 28* (*COR28*) gene of accessions in southern areas was associated with long-period and late-flowering phenotypes (Rees et al., 2021). Latitudinal clines of FRP in wild monkey flower (*Mimulus guttatus*) and cultivated soybean (*Glycine max*) were also reported (Greenham et al., 2017). During the domestication of tomato, an allele of the *EMPFINDLICHER IM DUNKELROTEN LICHT 1* (*EID1*) gene for a longer FRP was selected because of its higher crop performance under long-day conditions (Müller et al., 2016). These results imply that FRP variation is linked to local adaptation of clock-dependent temporal traits. However, direct evidence of the association between FRP and adaptive temporal traits is still limited because of difficulties in evaluating the latter in natural populations.

As a clock-dependent physiology in plants, photoperiodic flowering is a typical target of local adaptation; the critical day length (CDL) often shows a latitudinal cline to adjust the flowering time to local habitat conditions (Ray and Alexander 1966; Imamura et al., 1966; Katayama 1977; Yukawa and Takimoto, 1976). In *Arabidopsis*, the *CONSTANS* (*CO*) gene plays a key role in photoperiodism (Suárez-López et al., 2001). According to the external coincidence model, its diel expression pattern is critical in CDL determination, and the coincidence of light with *CO* expression activates the *FLOWERING LOCUS T* (*FT*) gene, the master regulator of floral induction. A clock-related gene, *GIGANTEA* (*GI*), is responsible for the expression pattern of *CO* (Sawa et al., 2007). Notably, an *Arabidopsis* clock gene mutant with a shorter FRP exhibited an early flowering phenotype under short-day conditions. A shorter FRP resulted in earlier *CO* induction, which broadened the coincidence window to induce *FT* expression earlier (Yanovsky and Kay, 2002). Thus, the induction timing of *FT* is a potential target for the local adaptation of CDL. Consistent with this idea, *Populus trichocarpa* showed a latitudinal cline in flowering and growth cessation, and the induction timing of *PtFT1* was earlier in the southern populations (Böhlenius et al., 2006). Because the natural variation of FRP is expected to be related to clock phase variation, we hypothesize that FPR-dependent phase changes in photoperiodic genes alter the coincidence window for *FT* activation and contribute to the local adaptation of CDL.

To investigate the relationship between FRP and CDL, we focused on the short-day duckweed *Lemna aequinoctialis*. Duckweed species are small, free-floating aquatic plants that have been studied in diverse fields ranging from ecology to genomics (Acosta et al., 2021). High-throughput phenotyping using the particle bombardment transfection technique and a luciferase reporter can be used in duckweed species to study the variation in circadian traits (Miwa et al., 2006; Muranaka et al., 2015; Isoda et al., 2022). A latitudinal cline of the CDL for photoperiodic flowering has been described in *L. aequinoctialis* Japanese populations (Yukawa and Takimoto, 1976). *L. aequinoctialis* produces flower buds within one week of photoperiodic treatment, allowing rapid CDL determination. Isolated duckweed strains are maintained by clonal growth, and laboratory experiments using clonal strains have enabled the precise determination of multiple phenotypic traits of the original genotypes from natural populations. By taking advantage of this plant material, we detected a significant correlation between FRPs and locally adaptive CDLs.

## Results

### Strain collection from natural populations in Japan

**Figure 1:**
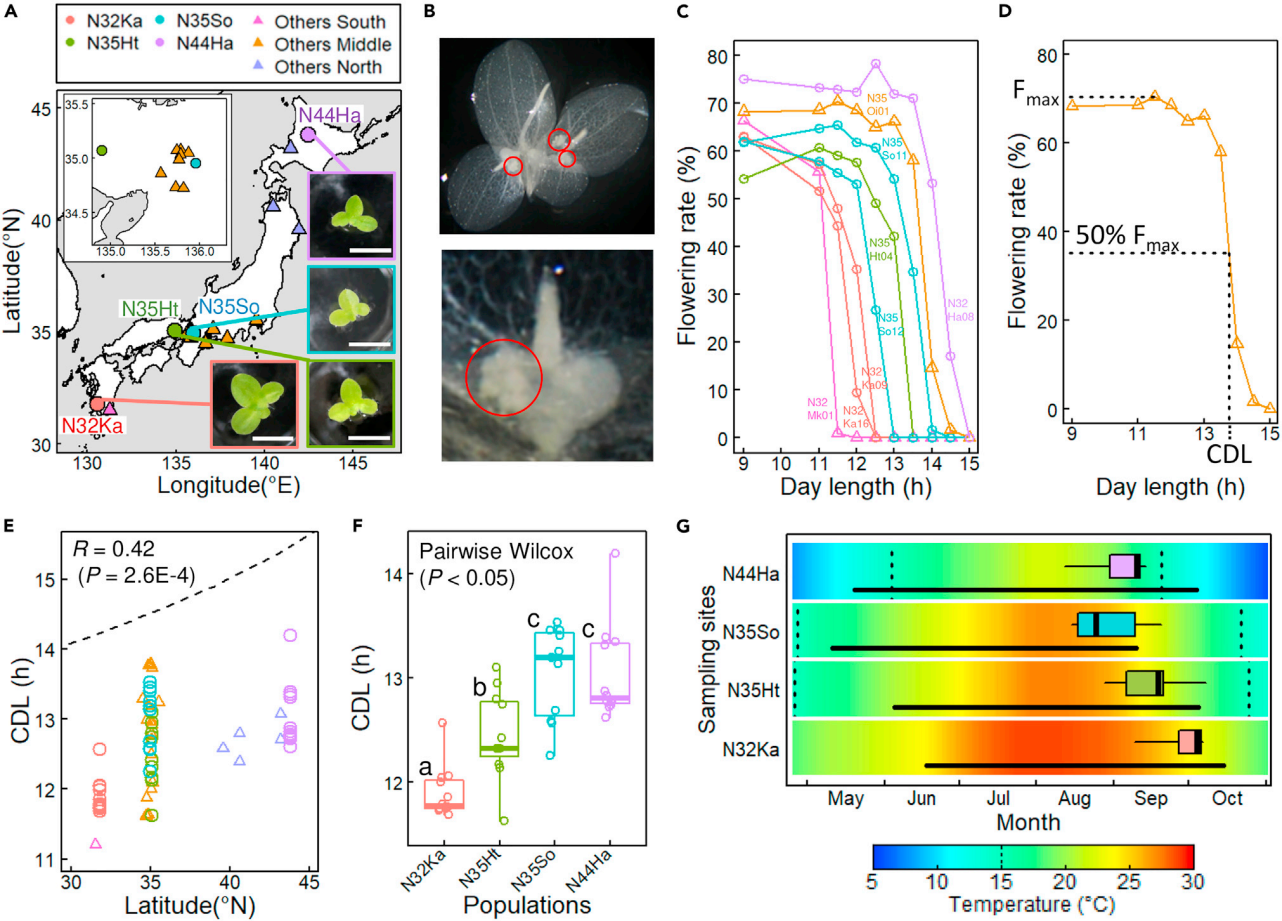
Local adaptation of critical day-length (CDL) in *Lemna aequinoctialis* strains isolated in Japan (A) The locations from which 11–12 strains (circles) and one to three strains (triangles) were isolated. The same colors and symbols apply to other panels. Examples of the strains in the four populations are shown. Scale bars: 5 mm. (B) A colony with floral buds (top). Colony of N35Oi03 strain bleached by EtOH after one week of short-day (9L15D) treatment and imaged from the underside. Close-up underside view around the meristematic tissue. Red circles indicate floral buds. (C) Continuous variation of photoperiodic response on flowering. The flowering rate after one week of photoperiodic treatment is plotted against day-length. Eight strains in six populations are shown. (D) Critical day length (CDL) was determined as the day length where 50% of the maximum flowering rate (Fmax) is expected. See STAR Methods for more detail. (E) Latitudinal cline of CDL. The Pearson’s correlation coefficient and p-value are shown. The dashed line represents the day-length of the summer solstice. (F), CDL variation among the four populations. Boxplots with points representing individual strains are shown. Different letters indicate significant differences based on the pairwise Wilcoxon test (p < 0.05). (G) Timing of CDLs in the year at four sampling sites. The dates for CDLs shown in panel C are represented as a boxplot at each site. Black horizontal lines represent the flooding period. The background colors represent a 30-year (1990–2019) mean of daily temperature. Vertical dotted lines represent 15°C.

We collected 72 strains of *L. aequinoctialis* from 20 populations between latitudes 31.5°N and 43.8°N (Figure 1A; Table S1). To estimate both intra- and inter-population variations, 11–12 strains were collected from four populations (N32Ka, N35Ht, N35So, and N44Ha). The strains were aseptically maintained by clonal growth. The frond size showed intra- and inter-population variation, suggesting heterogeneous genotypes, even within a population (Figure S1). The estimated genome sizes of all strains were similar (Figure S2).

### Natural variation in CDLs

These strains detected day lengths with a resolution of 0.5 h, and their CDLs ranged from 11.2 to 14.2 h (Figures 1B, 1C, and 1D). As previously reported (Yukawa and Takimoto, 1976), a latitudinal cline was observed in CDLs: 11.2–12.6 h in the south, 12.4–14.2 h in the north, and a large variation in CDL was observed at approximately 35°N (Figure 1E). The longer CDL at higher latitudes is consistent with that of other short-day flowering species (Ray and Alexander 1966; Imamura et al., 1966; Katayama 1977). Intrapopulation differences of 1–1.5 h in CDLs were observed in each population of N32Ka, N35Ht, N35So, and N44Ha (Figure 1F). The significant differences between these populations suggest a geographical differentiation of CDLs. The difference in CDL between the two 35°N populations, N35Ht and N35So, suggests that the adaptive CDL at each site may differ, even at the same latitude (Figure 1F). The flooding period of the paddy fields differed between the sampling sites depending on the cultivation schedule of the different rice cultivars. The longer CDL of N35So, corresponding to earlier flowering, is consistent with the earlier cessation of flooding at this site than at the N35Ht site (Figure 1G). The CDL of the N32Ka population was shorter than that of the N35 population (Figure 1F). The CDLs of the three populations appear to be linked to the cultivation schedule at each site (Figure 1G). The complete drainage of paddy fields during and after rice harvesting is a potential selection pressure for duckweed (Natuhara, 2013; Fujita et al., 2015). Although the rice harvesting time at the N44Ha site was comparable to that at the N35Ht site (Figure 1F), the CDL of N44Ha was longer than that of N35Ht. At the N44Ha site, the low-temperature season (<15°C: lower limit for *L. aequinoctialis* growth) began before the rice harvest time (Landolt, 1986), suggesting that low temperatures, rather than rice harvest time, might be a potential selection pressure. Overall, taking these various considerations into account, the CDLs of the four populations were likely to fit a suitable season for reproduction at each site.

### Natural variation in circadian rhythms

**Figure 2:**
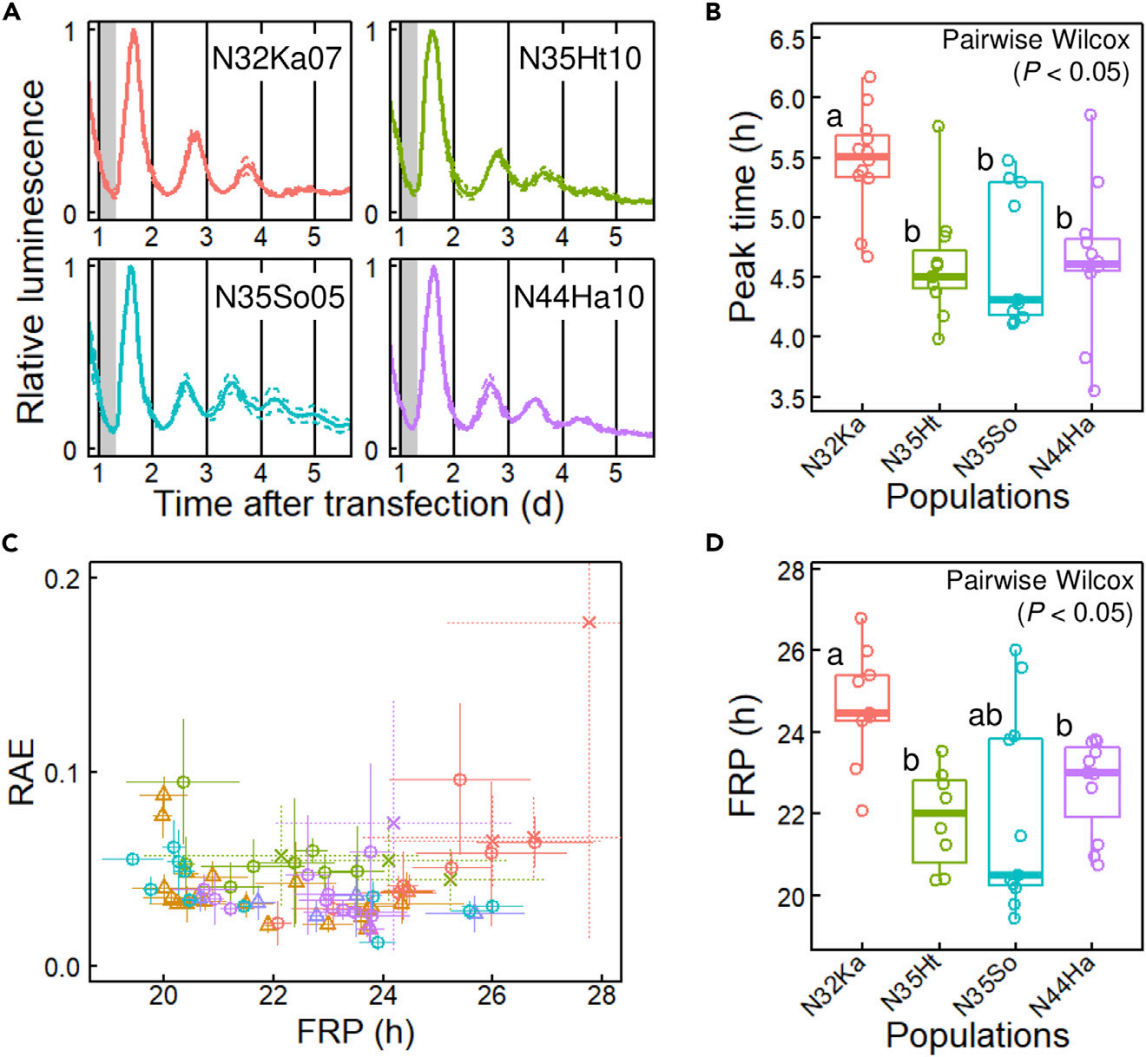
Natural variation of circadian rhythms in *Lemna aequinoctialis* strains (A) Examples of luminescence rhythms of *AtCCA1::LUC*. Solid and dashed lines represent the mean and ±SD of three replications, respectively. Gray boxes indicate the dark period. The strain name is indicated in each plot. (B) Variation of the timing of the first peak in constant light (LL) among the four populations. Boxplots with points for individual strains are shown. Different letters indicate significant differences based on the pairwise Wilcoxon test (p < 0.05). (C) Relative amplitude error (RAE) of each strain plotted against its free-running period (FRP). The mean values of three replications are shown. Error bars = SD The crosses represent seven strains that showed unstable rhythms (SD of FRP >1.5 h, Figure S3). Population names are represented in the graph legend. (D) Variation of the free-running period (FRP) in LL among the four populations. Boxplots with points for individual strains are shown. Seven strains with unstable rhythms (SD of FRP >1.5 h) were excluded from the plot. Different letters indicate significant differences based on the pairwise Wilcoxon test (p < 0.05).

Natural variation in circadian rhythms was assessed using a luciferase reporter assay (Muranaka et al., 2015). Using particle bombardment, a luminescent reporter, *AtCCA1::LUC* (Nakamichi et al., 2004), was semi-transiently introduced into plants grown under long-day conditions (15L9D; 15-h light/9-h dark). The plants were placed under an automatic luminescent monitoring system and released into constant light (LL) after two days of 15L9D (Figure 2A). All strains showed clear diel luminescence rhythms with morning peaks at 15L9D. Among the four populations, the peak time of N32Ka was significantly later than those of the other three populations (Figure 2B). To estimate FRP under LL, a fast Fourier transform-nonlinear least squares (FFT-NLLS) analysis was used (Zielinski et al., 2014), in which the rhythm significance was estimated by a relative amplitude error (RAE) that increased from 0 to 1 as the rhythm neared statistical insignificance (Figure 2C). Seven strains with a high RAE value (>0.1) or a high SD of FRP (>1.5 h) were excluded from the subsequent analysis (Figure S3). The FRP of N32Ka was significantly longer than those of the other populations, corresponding to its late peak time (Figures 2B and 2D). This is consistent with the idea of an FRP-dependent phase phenotype, that is, a positive correlation between FRP and peak time (Helm et al., 2017).

### A negative correlation between FRP and CDL

**Figure 3:**
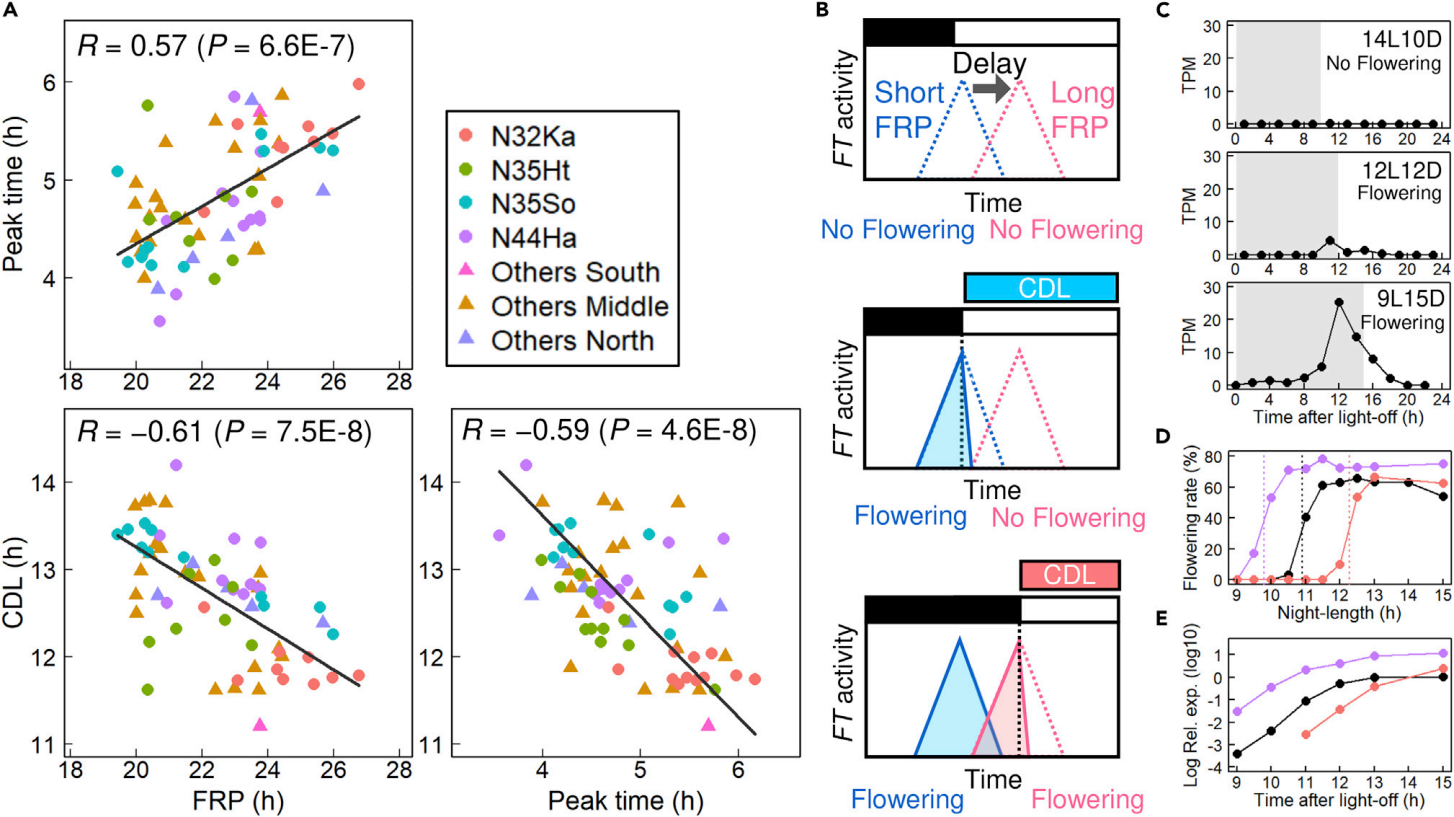
The effect of FRP-dependent phase phenotype on photoperiodic flowering (A) The correlations between FRP, peak time, and CDL of *L. aequinoctialis* strains. The Pearson’s correlation coefficient and p-value are shown in each graph. The black lines represent Deming regression lines. Plots of seven strains with unstable rhythms (SD of FRP >1.5 h) are excluded from graphs that include FRP. (B) The hypothetical mechanism for the negative correlation between FRP and CDL. The *FT* induction is assumed to be gated by the circadian clock and permitted in the dark. The dotted lines and filled triangles schematically represent gate timing and the *FT* activity, respectively. The blue and red colors correspond to strains with a short and long FRP, respectively. (C) A photoperiodic response of the *LaFTh1* expression. Means of four or two RNA-seq experiments are plotted. The gray box indicates a dark period. The plots of individual experiments are shown in Figure S4C. (D) Photoperiodic responses of flowering for three strains (purple, N44Ha08; black, Nd; red, N32Ka06). The flowering rates are plotted against the night length of each photoperiod. Each strain’s critical night length (24 – CDL) is shown as a dashed line. (E) *LaFTh1* induction in the three strains during constant dark following 15L9D. The mRNA accumulation quantified by qPCR is plotted. The mRNA expression in N32Ka06 at 9 and 10 h were undetected. The colors are the same as in panel D.

Correlation analysis using data from all 72 strains (20 populations) suggested an effect of FRP-dependent phase phenotype on photoperiodism (Figure 3A). A positive correlation between FRP and peak time was observed at the genotypic level. In addition, CDL showed a negative correlation with both FRP and peak time. These results suggest that the variation in the FRP-dependent phase phenotype underlies the variation in the CDL. The CDL shift can be explained by an FRP-dependent phase shift of *FT* expression in the external coincidence model (Figure 3B). In this model, the *FT* induction is assumed to be gated by the circadian clock and permitted in the dark. Thus, *FT* expression responds to night lengthening. A longer FRP causes a phase delay in the gate timing and, consequently, a shorter CDL, resulting in a negative correlation between FRP and CDL.

### Diel expression patterns of *FT*, *CO,* and *GI* homologs

We obtained diel transcriptomes of *L. aequinoctialis* pure line (Nd strain, Muranaka et al., 2015) under three photoperiod conditions to explore *FT* homologs. The *de novo* assembly detected five *FT* homologs. They were named *LaFTh1–5* (Figures S4A and S4B). In a phylogenetic tree with FT homologs of various plant species, LaFTh1 composes a clade with a *Zea mays* FT homolog (ZmZCN8) and a *Brachypodium distachyon* FT homolog (BdFTL9). *LaFTh1* was induced before the night’s end, and its expression increased during longer-night conditions (Figures 3C and S4C). Interestingly, it has been reported that *ZmZCN8* and *BdFTL9* are induced during the night only under short-day conditions (Meng et al., 2011; Qin et al., 2019). The transgenic *A. thaliana* carrying an *LaFTh1* overexpression construct showed an early flowering phenotype, suggesting its floral induction activity (Figure S4D). *LaFTh1* was induced even in the extended dark following a non-flowering long-day condition, suggesting that *LaFTh1* expression directly responds to night lengthening. The timing of *LaFTh1* induction differed among the three strains with different CDLs, and the variation in induction timing appeared to be responsible for the CDL variation (Figures 3D and 3E). Thus, the induction timing of *LaFTh1* is key for connecting the clock phase to photoperiodism at the molecular level. Notably, the *LaFTh1* expression levels showed a large difference. *LaFTh1* expression in the N44Ha08 strain was 10-fold higher than in the other two strains (Figure 3E). This high basal level may have caused the extremely long CDL of 14.2 h (Figure 1F).

Next, we explored the *L. aequinoctialis* homologs of *CONSTANS* (*CO*) and *GIGANTEA* (*GI* ), which are core components of day-length measurement, by regulating *FT* expression in *Arabidopsis* photoperiodism (Sawa et al., 2007). We detected three *CO* homologs (*LaCOh1-3*) with amino acid sequence similarities to AtCOL3 and AtCOL4, rather than AtCO (Figure S5A). The *LaCOh1-3* genes showed diel expression patterns, but none had the midnight peak phase observed for *AtCO* and *OsHd1* (the rice *CO* ortholog) (Yanovsky and Kay 2002; Hayama et al., 2003). In contrast, a *GI* homolog (*LaGIh1*) showed diel expression patterns that were similar to those of *Arabidopsis* and rice (Figure S5B) (Sawa et al., 2007; Hayama et al., 2003). These results suggest that the molecular mechanism of day-length measurement in *L. aequinoctialis* is different from that in *Arabidopsis* and rice.

## Discussion

Our study demonstrated the effect of the FRP-dependent phase phenotype on locally adapted traits; CDL variation is tightly coupled with FRP variation, even in natural populations. Diel transcriptome analysis suggests that *FT* induction timing, which is likely to be related to FRP-dependent phase variation, is a determining factor for CDL and, consequently, the flowering season. These results indicated the importance of circadian phenotypes in the natural selection of seasonal phenotypes. This study also provides new insights into the local adaptation of CDLs to paddy field environments. Consistent with previous studies using short-day plants, the populations of *L. aequinoctialis* in Japan showed a latitudinal cline in their CDLs, suggesting that the flowering season of this plant adapted to the local climate, exhibiting a latitudinal cline in temperature (Hut et al., 2013). In addition to the latitudinal cline, another factor in the local adaptation of CDLs should be considered to explain the variation of CDLs among populations at similar latitudes (Figure 1E). Interestingly, the CDLs appeared to adapt to the flooding season at each sampling site (Figure 1G). The improved drainage systems associated with the agricultural modernization of Japan that began in the 1950s have resulted in dry paddy field environments in winter and consequently altered the weed flora (Katayama et al., 2015). The natural variation of the CDLs possibly contributed to the adaptive evolution of the flowering season in response to the rapid artificial shift of the flooding season. In addition to interpopulation variations, CDLs showed intrapopulation variations (Figure 1F). The flooding period of paddy fields is highly dependent on the rice cultivar used. Rice cultivars planted in the same area may be spatially or yearly heterogeneous. Such artificial fluctuations may promote intrapopulation variation in CDLs. The natural variation in FRP-dependent phase phenotypes may play a role in maintaining CDL variations. In *A. thaliana*, the recombinant inbred lines of two accessions with similar FPRs showed variation as substantial as that observed in the global collection (22.0–28.5 h) (Michael et al., 2003). Such transgressive segregation of FPR is caused by the complex interference among many quantitative loci (Rieseberg et al., 2003), and may play an important role in providing phenotypic variations for rapid evolutionary adaptation to irregular environmental changes (Hamann et al., 2021). In this respect, the apparent tetraploidy of *L aequinoctialis* (Figure S2; Beppu and Takimoto, 1981; Wang et al., 2011) could have contributed to the generation of phenotypic variation by increasing the rates of evolution and number of components in gene regulatory networks (Sémon and Wolfe 2007; Selmecki et al., 2015).

**Figure 4:**
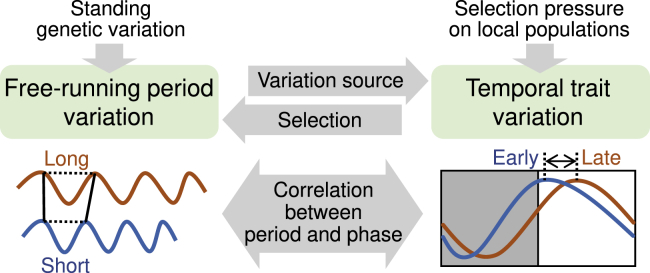
The effect of the FRP-dependent phase shift on local adaptation of temporal traits FRP variation functions as a resource for the variation of temporal traits in the process of local adaptation. Genetic variations in many quantitative loci for FRP are selected based on their phenotypic effect on temporal traits under day-night conditions. In *L. aequinoctialis*, the selection pressure for early flowering phenotype appeared to select a short-FRP genotype related to its early induction timing of an *FT* gene.

As the circadian clock regulates various aspects of physiology (Greenham and McClung, 2015; Helm et al., 2017), FRP variation potentially contributes to the fine-tuning of many different traits in the process of local adaptation. Conversely, selection pressure on clock-dependent physiological traits may favor mutations in clock-related genes, which alter FRP, and consequently, the diel phase (Figure 4). This is a possible reason for the maintenance of FRP variation with standing genetic variations in clock-related genes in the natural populations (Hut et al., 2013; Salmela and Weinig, 2019). Thus, the adaptive significance of FRP variation should be considered in relation to the phase phenotype under day-night conditions. Interestingly, a latitudinal cline of FRP has been reported in the linden bug *Pyrrhocoris apterus*, in which circadian clock genes are responsible for the photoperiodic response of the reproductive diapause (Pivarciova et al., 2016; Urbanová et al., 2016). FRP-dependent phase variation may be involved in local adaptation in various organisms. Longer FRP in soybean and tomato appears to be associated with higher crop performance under long-day conditions (Müller et al., 2016; Greenham et al., 2017). The FPR of the circadian clock is a potential target for crop improvement (Steed et al., 2021). The circadian clock was acquired for adaptation to day-night cycles and has evolved as a hub of environmental response systems involving multiple input pathways (Dixon et al., 2014). As a result, the circadian system can function as a source of variation for temporal traits, resulting from many quantitative loci for FRP in the process of adaptive evolution (Nagel and Kay, 2012; Boyle et al., 2017; Sun et al., 2021).

### Limitations of the study

Although *L. aequinoctialis* is a good model for investigating the association between FRP and CDL, the understanding of the molecular basis of photoperiodism in this plant is insufficient to directly verify the effect of the FRP-dependent phase phenotype on CDL determination at the molecular level. To access the genetic basis of the natural variation of FRP and CDL, functional analysis of homologs of the circadian clock, light signaling, and photoperiodism genes is required. We should note the importance of factors other than FRP in CDL determination because the high basal level of *LaFTh1* appears to contribute to the longer CDL in the northern strain. Future studies should provide genomic information and genetic tools for *L. aequinoctialis*. In addition to molecular analysis, an ecological field study is required to confirm CDL adaptation to the flooding period in paddy fields.

## STAR★Methods

### Key resources table

**Table:** 

REAGENT or RESOURCE	SOURCE	IDENTIFIER
**Chemicals, peptides, and recombinant proteins**		

D-luciferin potassium salt	Fujifilm Wako Chemicals	126–05116
RNAlater	Sigma Aldrich	R0901
Streptavidin Magnetic Beads	New England Biolabs	S1420S
RevertAid Reverse Transcriptase	Thermo Fisher	EP0441
DNA polymerase I	Thermo Fisher	EP0041
AMpure magnetic beads	Beckman Coulter	A63881

**Critical commercial assays**		

Partec CyStain UV Precise P reagent kit	Sysmex Partec GmbH	AF009546
NucleoSpin RNA Plant	MACHEREY-NAGEL GmbH & Co	740949.50
ReverTra Ace qPCR RT Master Mix	TOYOBO	FSQ-201
THUNDERBIRD SYBR qPCR Mix	TOYOBO	QPS-101
KAPA HiFi HS ReadyMix	Kapa Biosystems	7958927001

**Experimental models: Organisms/strains**		

*Lemna aequinoctialis* 72 strains: N44Ha01, N44Ha02, N44Ha03, N44Ha04, N44Ha05, N44Ha08, N44Ha09, N44Ha10, N44Ha12, N44Ha13, N44Ha15, N44Ha17, N43Hi01, N43Hi02, N41Ac01, N41Ac02, N40Im01, N36Ky01, N35At01, N35Ki01, N35Ki02, N35Ht01, N35Ht02, N35Ht04, N35Ht05, N35Ht06, N35Ht07, N35Ht08, N35Ht09, N35Ht10, N35Ht11, N35Ht12, N35Kk01, N35Sb01, N35Sb02, N35Sb03, N35Kn01, N35Kn04, N35Kn09, N35Kr01, N35So01, N35So02, N35So03, N35So04, N35So05, N35So06, N35So08, N35So10, N35So11, N35So12, N35So15, N35Oi01, N35Oi02, N35Oi03, N35Nn01, N35Nn02, N35Nk01, N35Si01, N34Mi01, N32Ka01, N32Ka02, N32Ka04, N32Ka06, N32Ka07, N32Ka08, N32Ka09, N32Ka10, N32Ka11, N32Ka14, N32Ka16, N32Ka18, N32Mk01	This paper	N/A
*Lemna aequinoctialis* strain: ND	Muranaka et al., 2015	N/A
*Lemna aequinoctialis* strain: 6746	Muranaka et al., 2015	N/A
*Lemna gibba* strain: p8L	Muranaka et al., 2015	N/A
*Arabidopsis thaliana* strain: Col-0		

**Oligonucleotides**		

qPCR Primer: LaFTh1_fwd ACCCTACCCTTAGAGAATATCTGC	This paper	N/A
qPCR Primer: LaFTh1_rev TAGGTGCTGGGCCTTCATAG	This paper	N/A
qPCR primer: LaACT2_fwd ACACAGTGCCCATCTATGAAGG	This paper	N/A
qPCR primer: LaACT2_fwd AGTAGCCTCGTTCGGTTAGGATC	This paper	N/A
Cloning primer: LaFTh1_fwd CGCGGCCGCCACCATGACACCTCAGGATCCCTTGTG	This paper	N/A
Cloning primer: LaFTh1_rev CGGCGCGCCCTAGGTAAACCGCCTTCCACCAG	This paper	N/A

**Deposited data**		

RNA-seq data	DDBJ	PRJDB12719
cDNA of LaFTh1	DDBJ	LC662606

**Recombinant DNA**		

Plasmid: *pUC-AtCCA1::LUC+*	Nakamichi et al., 2004	N/A
Plasmid: pENTR-D/TOPO vector	Karimi et al., 2002 Invitrogen	K240020
Plasmid: pK7WG2 overexpression binary vector	Zhang et al., 2006	N/A

**Software and algorithms**		

ImageJ (Fiji)	Schneider et al., 2012	1.53c
R	Ihaka and Gentleman, 1996	3.6.3
Trimmomatic	Bolger et al., 2014	0.39
Trinity	Grabherr et al., 2011	2.8.4.
RSEM	Li and Dewey, 2011	1.3.1
bowtie2	Langmead and Salzberg, 2012	2.4.2
tblastn	Altschul et al., 1990	2.11.0+

**Other**		

RNA-seq was performed based on the BrAD-seq protocol.	Townsley et al., 2015	N/A

### Resource availability

#### Lead contact

Further information and requests for resources and plasmids should be directed to and will be fulfilled by the Lead Contact, Tokitaka Oyama (oyama.tokitaka.8w@kyoto-u.ac.jp).

#### Materials availability

This study did not generate new unique materials or reagents.

### Experimental model and subject details

#### Plant materials and growth conditions

Seventy-two *L. aequinoctialis* strains were collected from 20 sites in Japan (Table S1). The collected plants were sterilized by washing with 5% sodium hypochlorite for several minutes, followed by washing with sterilized water. From the successfully sterilized plants, a single colony was isolated. Only one strain was isolated from a paddy field, and a maximum of 12 paddy fields were selected at each site. These strains were maintained aseptically in a growth medium (NF medium containing 1% sucrose) under long-day conditions (15L9D; approximately 50 μmol m^−2^ s^−1^) in an incubator (LH350-SP, NK system, Japan) at 25 ± 1°C (Muranaka et al., 2015) *L. aequinoctialis* 6746 and *Lemna gibba* p8L strains were maintained in the same conditions. Before the experiments, the plants were grown in a flask or 6-well plastic plate filled with 30 mL or 8 mL of growth medium, respectively.

### Method details

#### Genome size estimation

The relative genome sizes of *L. aequinoctialis* strains were estimated based on 4’,6-diamidino-2-phenylindole diacetate dye flow cytometry (DAPI-FCM) using a Partec CyStain UV Precise P reagent kit (Sysmex Partec GmbH, Germany) and a flow cytometer (Partec Ploidy Analyzer PA-I, Sysmex Partec GmbH). *Lemna gibba* p8L was used as the standard. The relative genome sizes of th*e L. aequinoctialis* strains were calculated as the relative peak positions. The genome size of *L. gibba* has been estimated to be 486 Mbp (Wang et al., 2011).

#### Frond-length measurements

The top view image of the plants was captured using a digital camera (EOS 5D mark3 [Canon, Japan] with SP 90 mm Di MACRO [Tamron, Japan] or EOS Kiss X5 with EF-S55-250 mm [Canon]). The frond length was manually measured as the length from the base to the apex of the mother frond using ImageJ 1.53c software (Figure S1A). The frond lengths of the eight colonies were measured for each strain.

#### Measurement of the critical day length in photoperiodic flowering

A colony consisting of three or four visible fronds was placed in each well of a 24-well plastic plate filled with 2 mL of growth medium. These plants were subjected to a one-week photoperiodic treatment with day lengths of 9, 11, 11.5, 12, 12.5, 13, 13.5, 14, 14.5, and 15 h in an eight-chamber incubator (LH30-8-CT, NK system, Japan). The temperature was maintained at 25 ± 1°C and the light intensity was approximately 30 μmol m^−2^ s^−1^. After treatment, the plants were bleached with 70% ethanol. The number of flowering and non-flowering fronds was counted for each colony under a stereoscopic microscope. Flowering rate was calculated as the percentage of flowering fronds to the total number of fronds in at least two replicates. The critical day length was determined as the day length where 50% of the maximum flowering rate (Fmax) was expected in a piecewise linear function for the obtained flowering rate (Figure 1D).

#### Meteorological data

Temperature data at each site were obtained from the Automated Meteorological Data Acquisition System (AMeDAS) data download service (https://www.data.jma.go.jp/risk/obsdl/index.php). Day lengths were calculated using the online service of the National Astronomical Observatory of Japan (https://eco.mtk.nao.ac.jp/cgi-bin/koyomi/koyomix_en.cgi).

#### Monitoring luminescence rhythms

Luminescence monitoring using a circadian luminescent reporter was performed as follows (Muranaka et al., 2015). Plasmid DNA carrying the luciferase reporter gene *pUC-AtCCA1::LUC+* (*AtCCA1::LUC,* Nakamichi et al., 2004) was coated on 0.48 mg of gold particles (1.0 mm diameter, Bio-Rad) and introduced into plants laid on a 60-mm plastic dish using a particle bombardment system (PDS-1000/He, Bio-Rad) according to the manufacturer’s instructions (vacuum, 27 mmHg; helium pressure, 450 psi). After particle bombardment, the plants were divided into three 35-mm dishes (approximately four colonies per dish) filled with 4 mL of growth medium containing D-luciferin (0.2 mM potassium salt, Wako). A luminescence dish-monitoring system with photomultiplier tubes (H7360-01; Hamamatsu Photonics K.K., Japan) was used for the luminescence measurements. To reduce the background chlorophyll fluorescence, a short-pass filter (SVO630; Asahi Spectra, Japan) was set at the detection site of the photomultiplier tubes. Each dish was subjected to 30 s of measurements every 20 min. The monitoring system was placed in an incubator (KCLP-1000I-CT; NK System, Japan) with fluorescent lamps (FL20SSW/18; Mitsubishi Electric Co., Japan). The temperature was maintained at 25 ± 1°C and the light intensity was approximately 30 μmol m^−2^ s^−1^.

#### Time-series analysis

Peak detection and FRP estimation were performed as follows (Muranaka and Oyama, 2016). The peak positions were estimated by local quadratic curve fitting. For period estimation, the obtained luminescence time series were detrended by subtracting the 24-h moving average. Thereafter, the amplitude was normalized by dividing by the 24-h moving standard deviation. The normalized time series of 60 to 132 h was analyzed using fast Fourier transform-nonlinear least squares (FFT-NLLS) to determine the FRP (Zielinski et al., 2014). FFT-NLLS was based on a multicomponent cosine fit. Rhythm significance was estimated by a relative amplitude error (RAE) that increases from 0 to 1 as the rhythm approached statistical insignificance. These analyses were performed using R scripts developed and run with R 3.6.3 (http://r-project.org/).

#### RNA-seq analysis

Th*e L. aequinoctialis* Nd strain (6^th^ generation of self-fertilization of the N35Kn04 strain, Muranaka et al., 2015) was used for RNA-seq analysis. Plants were maintained under 15L9D conditions (approximately 50 μmol m^−2^ s^−1^, white LED T5LT20W, Beamtec, Japan) in an incubator (HCLP-880, NK system, Japan) at 25 ± 1°C. For the 14L10D and 12L12D experiments, the plants were divided into 48 x 35-mm dishes (four colonies per dish) with 4 mL growth medium per dish. After one week of photoperiodic treatment (14L10D or 12L12D), plants in each dish were collected every 2 h for 24 h (four replicates per sampling time). For the 9L15D experiment, plants were pre-cultured under 9L15D conditions for one week. Thereafter, these plants were divided into 24 x 35-mm dishes (six colonies per dish) with 4 mL of growth medium per dish. After one week of photoperiodic treatment (9L15D), plants in each dish were collected every 2 h for 24 h (two replicates at each sampling time). The collected plants were wiped of moisture with paper, then immediately immersed in RNAlater solution (Sigma Aldrich) at each time point and stored at −20°C before RNA extraction. RNA-seq library preparation was performed based on the BrAD-seq protocol, as follows (Townsley et al., 2015). Plants were homogenized with a lysate buffer using a multi-bead shocker (MB755U, Yasui Kikai, Japan). mRNA was extracted from the lysate using magnetic streptavidin beads (New England Biolabs) with a biotin-20nt-20T oligo. The mRNA was subjected to heat fragmentation (94°C for 1.5 min) and converted to cDNA using RevertAid Reverse Transcriptase (Thermo Fisher) with a 3-prime priming adapter with a random octamer sequence. The 5-prime adapter was then added to the cDNA and captured by the terminus of the RNA-cDNA hybrid. Its incorporation into a complete library molecule was catalyzed by DNA polymerase I (Thermo Fisher). The library was prepared using 16 (9L15D samples) or 18 (14L10D and 12L12D samples) PCR cycles with KAPA HiFi HS ReadyMix (Kapa Biosystems). The fragment size was selected to range from 300 bp to 600 bp using Ampure magnetic beads (Beckman Coulter). The quality of the library was checked using a bioanalyzer (Agilent). Paired-end 150 bp sequencing was conducted using the HiSeq system (Illumina). The obtained sequences (total 554 million reads for the 120 samples) were filtered using Trimmomatic v0.39 with option “LEADING:24 TRAILING:24 SLIDINGWINDOW:30:20 AVGQUAL:20 MINLEN:100” (Bolger et al., 2014). The filtered sequences were assembled *de novo* into 322,899 contigs (isoforms of 186,102 genes) using Trinity v2.8.4 (Grabherr et al., 2011). The 183,102 contigs (isoforms of 84,206 genes) that were longer than 400 bp were used as a reference sequence for mapping and read counting using RSEM v1.3.1 with bowtie2 v2.4.2 (Li and Dewey, 2011; Langmead and Salzberg, 2012). The mapped reads were 3.46 ± 1.22 million reads/sample (mean ± SD).

#### Detection of *FT, CO,* and *GI* homologs

*FT* homologs in the reference sequence (183,102 contigs) were searched using tblastn 2.11.0+ with a query for the OsHd3a amino acid sequence (UniProt-ID: Q93WI9). The top five *FT* homologs were named as *LaFTh1-5* based on their expression patterns and positions in the maximum likelihood phylogenetic tree with their estimated amino acid sequences (Figure S4B). LaFTh2 and LaFTh3 have the similar sequence as previously reported FT homologs, LaFTL3 and LaFTL1, respectively (Yoshida et al., 2021). *CO* homologs were searched with a query for the OsHd1 amino acid sequence (UniProt-ID: Q9SK53). The top three *CO* homologs were named as *LaCOh1-3* (Figure S5A)*. GI* homolog was searched with a query for the OsGI amino acid sequence (UniProt-ID: Q9AWL7) and named as *LaGIh1* (Figure S5B). A phylogenetic tree was constructed using the online tool ClustalW (https://www.genome.jp/tools-bin/clustalw) using the BioNJ algorithm. Colored multiple amino acid sequence alignments were generated using the online tool Clustal Omega (https://www.ebi.ac.uk/Tools/msa/clustalo/).

#### qPCR analysis

Eight to 12 colonies grown under 15L9D conditions were placed in a 35-mm dish with 4 mL of growth medium and maintained under the same conditions for a day, after which they were released to constant darkness at the end of 15 h of light and sampled every hour from 9 to 15 h after the light-off. The collected plants were wiped of moisture with paper, then immediately immersed in RNAlater solution (Sigma Aldrich) at each time point and stored at −20°C before RNA extraction. Total RNA was extracted using the NucleoSpin RNA Plant (MACHEREY-NAGEL GmbH & Co.) and converted to cDNA using ReverTra Ace qPCR RT Master Mix (TOYOBO). qPCR was performed using the StepOnePlus Real-Time PCR System (Applied Biosystems) with THUNDERBIRD SYBR qPCR Mix (TOYOBO). An *ACT2* homolog in *de novo* assembled sequences was determined using tblastn 2.11.0+ with a query of the OsACT2 amino acid sequence (UniProt-ID: A3C6D7) and used as a reference gene for the delta-delta Ct method. Primers for qPCR analysis were designed according to the *de novo* assembled sequences as follows: LaFTh1_fwd, 5’-ACCCTACCCTTAGAGAATATCTGC-3’; LaFTh1_rev, 5’-TAGGTGCTGGGCCTTCATAG-3’, LaACT2_fwd, 5’-ACACAGTGCCCATCTATGAAGG-3’; LaACT2_rev, 5’-AGTAGCCTCGTTCGGTTAGGATC-3’.

#### *LaFTh1* overexpression in *Arabidopsis thaliana*

To generate *LaFTh1* overexpressing transgenic *Arabidopsis* plants, the coding region of *LaFTh1* was amplified from cDNA derived from *L. aequinoctialis* Nd using the following primers: 5’-CGCGGCCGCCACCATGACACCTCAGGATCCCTTGTG-3’, and 5’-CGGCGCGCCCTAGGTAAACCGCCTTCCACCAG-3’. The amplified PCR fragment was digested with *Not*I and *Asc*I restriction enzymes and integrated into the pENTR-MCS cloning vector [pENTR-D/TOPO vector (Invitrogen) containing the pBluescript II multi-cloning sites] using the conventional linker ligation method. The sequence of *LaFTh1* CDS was transferred into the pK7WG2 overexpression binary vector (Karimi et al., 2002), which contained the *CaMV35S* promoter-driven expression cassette, using Gateway LR clonase II (Invitrogen). This construct was introduced into *Arabidopsis* wild-type plants Col-0 by the *Agrobacterium tumefaciens-*mediated floral dipping method (Zhang et al., 2006). Primary transformants (T1) were selected on 1x Murashige and Skoog culture media containing 1% sucrose, 0.8% agar, and 25 μg mL^−1^ kanamycin sulfate. Seeds were sterilized using chlorine gas, stratified for 3 days at 4°C, and then grown under short-day (10L14D) conditions to analyze the flowering phenotype in parallel with the selection process. Seedlings developing relatively long primary and lateral roots were regarded as kanamycin-resistant transgenic plants and transferred to a 1:1 mixture of vermiculite and soil (Nihon Hiryo Co., Ltd.). Several independent lines (*N* > 10) were isolated for further analysis of their morphological and flowering phenotypes. Most primary transformants could not produce mature siliques or seeds; however, some transgenic plants produced T2 seeds. Flowering phenotypes were also observed in the T2 generation.

### Quantification and statistical analysis

All boxplots in this manuscript display median lines, interquartile range boxes, and min/max whiskers. Different letters indicate significant differences based on the pairwise Wilcoxon test (p < 0.05). The pairwise Wilcoxon test was performed using the R built-in function “pairwise.wilcox.test” with *p* value adjustment using the Holm method. In correlation analysis, the Pearson’s correlation coefficient and p value was calculated by the R-built-in function “cor.test”.

## Data Availability

The RNA-seq raw sequences (PRJDB12719, https://ddbj.nig.ac.jp/resource/bioproject/PRJDB12719) and LaFTh1 CDS (LC662606, https://getentry.ddbj.nig.ac.jp/getentry/na/LC662606/) were deposited at DDBJ. The numerical data used for all plots (Data S1) and time-series data of luminescence (Data S2) are available in the supplementary information. This study did not generate any unique code.

## Supplemental information

Document S1. Figures S1–S5 and Table S1

Data S1. Summary of the experimental results for 72 strains, related to Figure 3 Supplied as EXCEL file

Data S2. Luminescence time series of 72 strains, related to Figure 2
