# Supplementary material for: Circadian-period variation underlies the local adaptation of photoperiodism in the short-day plant Lemna aequinoctialis
Source: iScience. 2022 Jun 17;25(7):104634. doi: 10.1016/j.isci.2022.104634 (PMC9253726; doi:10.1016/j.isci.2022.104634)
Supplement: Document S1. Figures S1–S5 and Table S1 [file mmc1.pdf]

iScience, Volume *25*

Supplemental information

Circadian-period variation underlies the local adaptation of photoperiodism in the short-day plant *Lemna aequinoctialis*

Tomoaki Muranaka, Shogo Ito, Hiroshi Kudoh, and Tokitaka Oyama

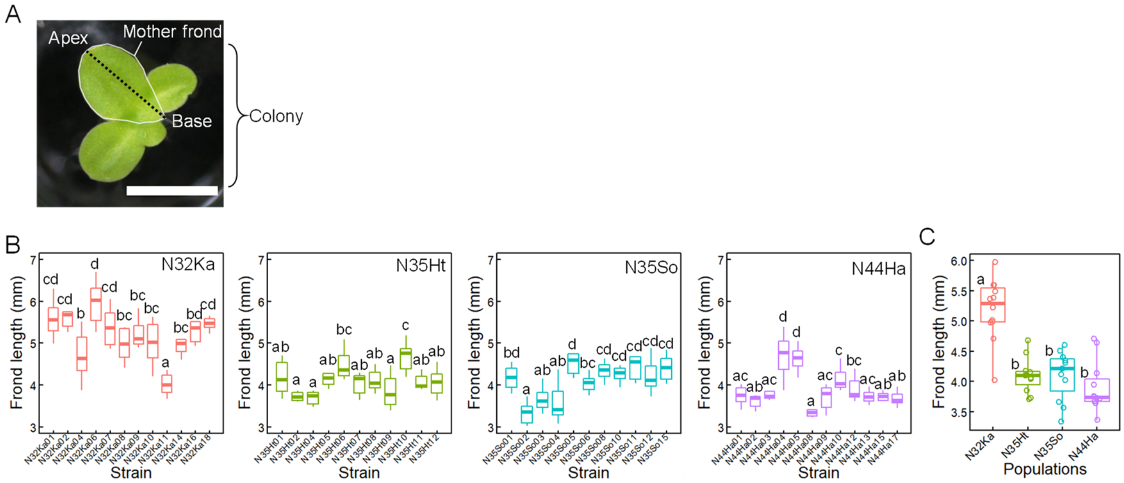

Figure S1. Intra- and inter-population variation of frond length in *L. aequinoctialis* strains, related to Figure 1. A, Definition of a frond length. The length of the mother frond (from base to apex, dotted black line) of eight colonies were measured for each strain of four populations (N32Ka, N35Ht, N35So, and N44Ha). Scale bar indicates 5 mm. **B,** Intrapopulation variation of frond length of the four populations are shown as boxplots. Different letters indicate significant differences based on Tukey's HSD test ($P < 0.05$). **C,** Interpopulation variation of frond length among the four populations. Different letters indicate significant differences based on pairwise Wilcoxon test ($P < 0.05$). Boxplots display a median line, interquartile range boxes, min/max whiskers.

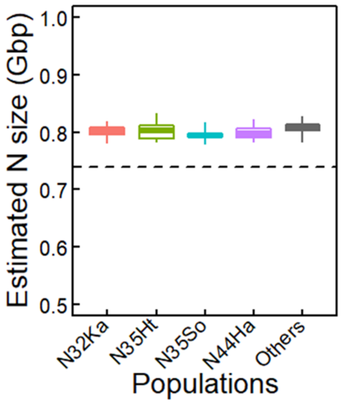

Figure S2. Similar genome sizes among 72 strains in different populations, related to Figure 1. Box plot displays a median line, interquartile range boxes, min/max whiskers of estimated genome sizes of the strains in a population (N32Ka, N35Ht, N35So, and N44Ha). "Others" includes 16 populations, which are indicated by triangles in Figure 1A. The genome size of a strain was estimated by flow cytometer. A dashed line represents the estimated genome size of *L. aequinoctialis* 6746 strain isolated in California, USA. No significant difference was detected based on pairwise Wilcoxon test.

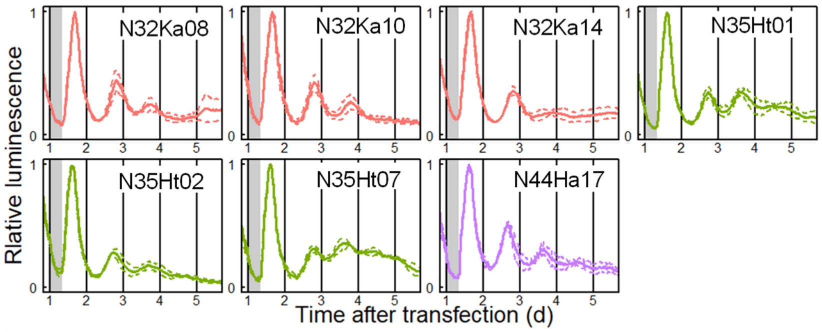

Figure S3. Luminescence rhythms of the seven strains excluded from the analysis, related to Figure 2. Luminescence intensity at each time point was normalized by the maximum intensity (first peak value). Solid and dashed lines represent mean and SD of three replications, respectively. Same populations are represented by the same colors.

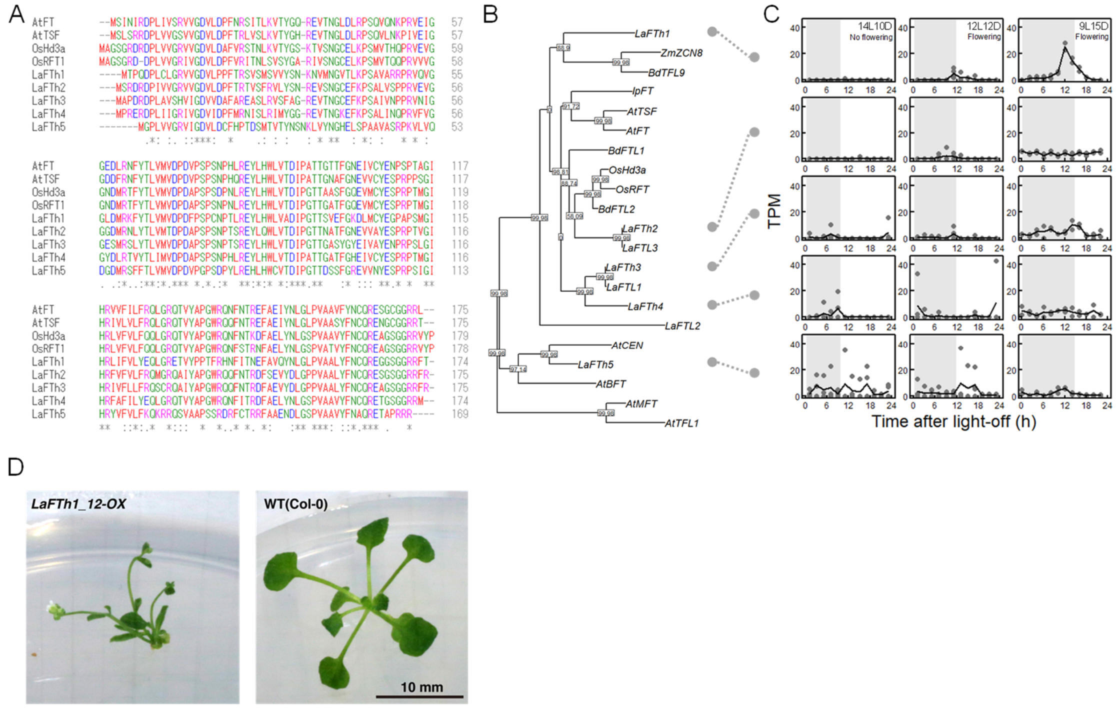

Figure S4. FT homologs of *L. aequinoctialis* Nd strain, related to Figure 3. A, Multiple amino acid sequence alignment of the five *L. aequinoctialis* FT homologs (LaFTh1-5) with the FT homologs of *Arabidopsis thaliana* (AtFT and AtTSF) and *Oryza sativa* (OsHd3a and OsRFT1). Colors represent physicochemical properties of residues. **B,** Maximum likelihood phylogenetic tree constructed with PhyML algorithm using full-length amino acid sequences. The LaFTL1-3 sequences were reported in Yoshida et al 2021. UniProt-IDs: A9LLY2: *Zea mays* ZCN8, I1HRJ5: *Brachypodium distachyon* FTL9, A8VJN3: *Ipomoea nil* FT, Q9S7R5: *Arabidopsis thaliana* TSF, Q9SXZ2: *A. thaliana* FT, I1HDB0: *B. distachyon* FTL1, Q93WI9: *Oryza sativa* Hd3a, Q8VWH2: *O. sativa* RFT, I1H0V9: *B. distachyon* FTL2, Q9ZNV5: *A. thaliana* CEN, Q9FIT4: *A. thaliana* BFT, Q9XFK7: *A. thaliana* MFT, Q2V822: *A. thaliana* TFL1. **C,** The expression patterns of five *FT* homologs (*LaFTh1* to *LaFTh5* from top to bottom) under three photoperiods (14L10D, 12L12D, 9L15D from right to left). The transcripts per kilobase million (TPM) values are plotted. Two (9L15D) or four (12L12D and 14L10D) experiments were performed for each photoperiodic condition. Black lines represent the mean values at each time point. Gray boxes indicate dark period. Three panels for *LaFTh1* expression pattern are plotted using the same dataset for the plots in Fig 3C. **D**, Early flowering phenotype of *Arabidopsis thaliana* carrying an *LaFTh1* overexpression construct. Plants of an *LaFTh1* overexpression line (T2 generation) (left) and Col-0 wild type (right) on an agar plate at 22 °C and 24 days (10L14D) after sowing.

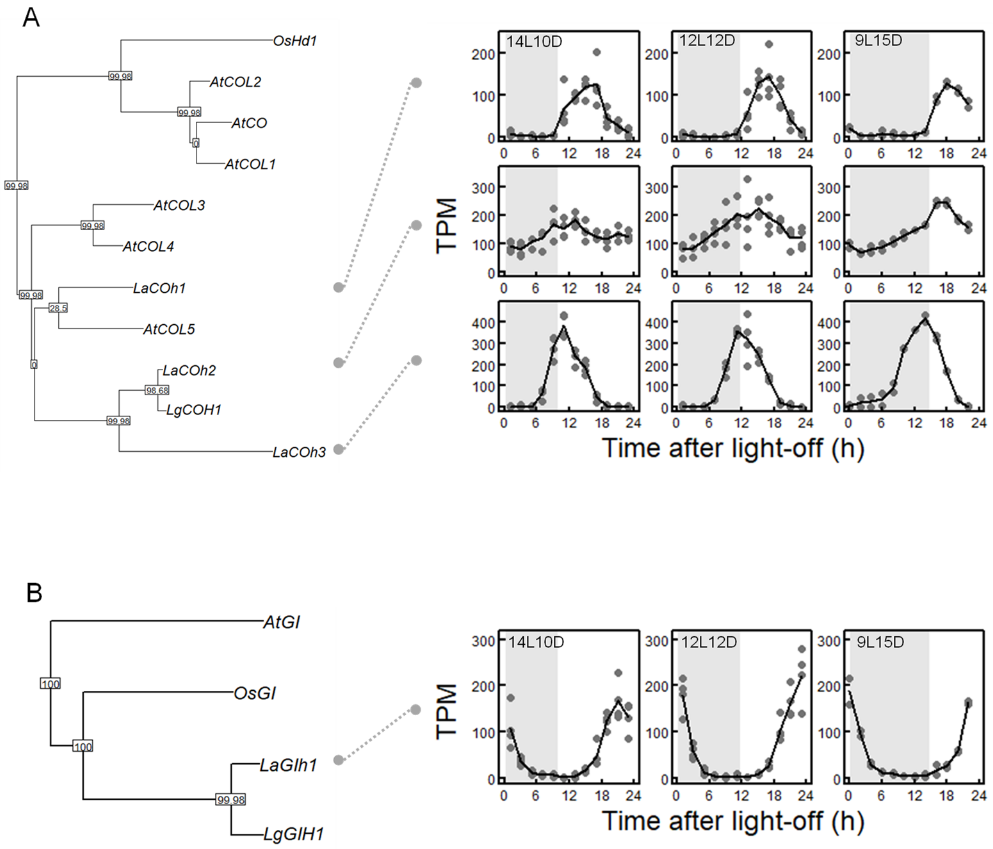

Figure S5. CO and GI homologs of *L. aequinoctialis* Nd strain, related to Figure 3. A, Maximum likelihood phylogenetic tree constructed with PhyML algorithm using full-length amino acid sequences of CO homologs, and the expression patterns of three *L. aequinoctialis CO* homologs (*LaCOh1*, *LaCOh2*, *LaCOh3* from top to bottom) under three photoperiods (14L10D, 12L12D, 9L15D from right to left). UniProt-IDs: Q9FHH8: *Arabidopsis thaliana* COL5, Q39057: *A. thaliana* CO, O50055: *A. thaliana* COL1, Q96502: *A. thaliana* COL2, Q9FDX8: *Oryza sativa* Hd1, Q9SK53: *A. thaliana* COL3, Q940T9: *A. thaliana* COL4, W0RZK9: *Lemna gibba* COH1 **B,** Maximum likelihood phylogenetic tree constructed with PhyML algorithm using full-length amino acid sequences of GI homologs, and the expression patterns of *LaGIh1* under three photoperiods (14L10D, 12L12D, 9L15D from right to left). The transcripts per kilobase million (TPM) values are plotted. Two (9L15D) or four (12L12D and 14L10D) experiments were performed for each photoperiodic condition. Black lines represent the mean values at each time point. Gray boxes indicate dark period. UniProt-IDs: Q9SQI2: *Arabidopsis thaliana* GI, Q50K77: *Lemna gibba* GIH1, Q9AWL7: *Oryza sativa* GI.

#	Population name	Symbol in Figs	Sampling site	Water system	Latitude	Longitude	Number of isolated strains
1	N44Ha	N44Ha	Asahikawa, Hokkaido	Paddy field	43.80468	142.45088	12
2	N43Hi	Others North	Ishikari, Hokkaido	Paddy field	43.1943	141.45465	2
3	N41Ac	Others North	Hirosaki castle, Aomori	Castle moat	40.60337	140.46717	2
4	N40Im	Others North	Miyako, Iwate	Paddy field	39.56776	141.93836	1
5	N36Ky	Others Middle	Yokohama, Kanagawa	Paddy field	35.52783	139.52045	1
6	N35At	Others Middle	Togo, Aichi	Paddy field	35.11062	137.083	1
7	N35Ki	Others Middle	Iwakura, Kyoto	Paddy field	35.07706	135.78781	2
8	N35Ht	N35Ht	Taka, Hyogo	Paddy field	35.07304	134.90231	11
9	N35Kk	Others Middle	Kyoto, Kyoto	Paddy field	35.07206	135.74302	1
10	N35Sb	Others Middle	Biwako, Shiga	Lake	35.04908	135.87446	3
11	N35Kn	Others Middle	Nougaku, Kyoto	Paddy field	35.03259	135.7836	3
12	N35Kr	Others Middle	Kamogawa river, Kyoto	Rivier	34.9896	135.76769	1
13	N35So	N35So	Otsu, Shiga	Paddy field	34.95615	135.95493	11
14	N35Oi	Others Middle	Ibaraki, Osaka	Paddy field	34.85884	135.56461	3
15	N35Nn	Others Middle	NAIST, Nara	Paddy field	34.7317	135.73142	2
16	N35Nk	Others Middle	Kizugawa, Nara	Paddy field	34.7242	135.8158	1
17	N35Si	Others Middle	Iwata, Shizuoka	Paddy field	34.72128	137.89284	1
18	N34Mi	Others Middle	Ise, Mie	Paddy field	34.49805	136.67711	1
19	N32Ka	N32Ka	Aira, Kagoshima	Paddy field	31.77734	130.56278	12
20	N32Mk	Others South	Kushimoto, Miyazaki	Paddy field	31.51068	131.25419	1

Table S1. Summary of twenty sampling sites, related to Figure 1.
